# 

**Published:** 1849-08

**Authors:** 


### BOOKS RECEIVED.

Through Messrs. Beckwith & Morton we have received from Messrs. Ticknor & Co., Boston, Tardieu's Treatise on Epidemic Cholera. From Messrs. Lea & Blanchard, Philadelphia, the great English work on Anatomy by the Drs. Quain & Sharpey; and the work of Dr. Tyler Smith on Parturition and the Principles and Practice of Obstetrics.

We have only space now to speak of one of these works: we allude to *Tardieu's Treatise on Cholera.* The author has prepared an excellent monograph on Epidemic cholera, which, though defective in some important particulars, is one that may be studied by the medical practitioner with great advantage. The historical department is not as full as it should be, but its details are very interesting and instructive. In the review of the causes of the epidemic there is much useful information, and the full length portrait of the disease itself is of the highest value. We cannot give that cordial approbation to the treatment that we should like to bestow on every part of the labors of the author. The plan of medication, of which we have given an outline sketch under the head of Progress of Cholera, is the one that has most thoroughly won our confidence. We feel persuaded that modifications of that treatment, made to suit the age, sex, temperament and peculiar condition of each patient, will be found eminently satisfactory to all intelligent physicians. No sensible practitioner will follow a routine treatment, but will suit his means to each particular case.

We take pleasure in commending Tardieu's Treatise to the study of our readers, and for the correct pathology of the disease, and its philosophical treatment, we commend the papers of Dr. Ayre, published in the December, January and February numbers of the *London Lancet.*

Of the other books, enumerated above, we shall take occasion to make an extended notice in the next No. of the Journal.

